# Association of Genetic Risks With Autism Spectrum Disorder and Early Neurodevelopmental Delays Among Children Without Intellectual Disability

**DOI:** 10.1001/jamanetworkopen.2019.21644

**Published:** 2020-02-05

**Authors:** Nagahide Takahashi, Taeko Harada, Tomoko Nishimura, Akemi Okumura, Damee Choi, Toshiki Iwabuchi, Hitoshi Kuwabara, Shu Takagai, Yoko Nomura, Nori Takei, Kenji J. Tsuchiya

**Affiliations:** Research Center for Child Mental Development, Hamamatsu University School of Medicine, Hamamatsu, Japan (Takahashi, Harada, Nishimura, Okumura, Choi, Iwabuchi, Nomura, Takei, Tsuchiya); Department of Psychiatry, Hamamatsu University School of Medicine, Hamamatsu, Japan (Kuwabara, Takagai); Queens College and Graduate Center, City University of New York, New York (Nomura).

## Abstract

**IMPORTANCE:**

Autism spectrum disorder (ASD) is highly heritable, and modest contributions of common genetic variants to ASD have been reported. However, the association of genetic risks derived from common risk variants with ASD traits in children from the general population is not clear, and the association of these genetic risks with neurodevelopment in infants has not been well understood.

**OBJECTIVE:**

To test whether a polygenic risk score (PRS) for ASD is associated with neurodevelopmental progress at age 18 months and ASD traits at age 6 years among children from the general population.

**DESIGN, SETTING, AND PARTICIPANTS:**

In this cohort study, 876 children in the Hamamatsu Birth Cohort for Mothers and Children in Hamamatsu, Japan, underwent testing for the association of an ASD PRS with neurodevelopmental progress and ASD traits. Data collection began in December 2007 and is ongoing. Data analysis was conducted from April to December 2019.

**MAIN OUTCOMES AND MEASURES:**

Summary data from the largest genome-wide association study were used to generate ASD PRSs, and significance of thresholds was calculated for each outcome. The Autism Diagnostic Observation Schedule 2 was used to measure ASD traits at age 6 years, and the Mullen Scales of Early Learning was used to measure neurodevelopmental progress at age 18 months.

**RESULTS:**

Of 876 participants (mean [SD] gestational age at birth, 38.9 [1.6] weeks; 438 [50.0%] boys; 868 [99.1%] Japanese), 734 were analyzed. The ASD PRS was associated with ASD traits (*R*^2^ = 0.024; β, 0.71; SE, 0.24; *P* = .03). The association of ASD PRS with infant neurodevelopment was most pronounced in gross motor (*R*^2^ = 0.015; β, −1.25; SE, 0.39; *P* = .01) and receptive language (*R*^2^ = 0.014; β, −1.19; SE, 0.39; *P* = .02) scores on the Mullen Scales of Early Learning. Gene set enrichment analyses found that several pathways, such as cell maturation (*R*^2^ = 0.057; β, −5.28; SE, 1.40; *P* < .001) and adenylyl cyclase activity and cyclic adenosine monophosphate concentration (*R*^2^ = 0.064; β, −5.30; SE 1.30; *P* < .001), were associated with ASD traits. Gene sets associated with inflammation were commonly enriched with ASD traits and gross motor skills (eg, chemokine motif ligand 2 production: *R*^2^ = 0.051; β, −6.04; SE, 1.75; *P* = .001; regulation of monocyte differentiation: *R*^2^ = 0.052; β, −6.63; SE, 1.90; *P* = .001; and B-cell differentiation: *R*^2^ = 0.051; β, 7.37; SE, 2.15; *P* = .001); glutamatergic signaling–associated gene sets were commonly enriched with ASD traits and receptive language skills (eg, regulation of glutamate secretion: *R*^2^ = 0.052; β, −5.82; SE, 1.68; *P* = .001; ionotropic glutamate receptor signaling pathway: *R*^2^ = 0.047; β, 3.54; SE, 1.09; *P* = .001; and negative regulation of glutamate secretion: *R*^2^ = 0.045; β, −5.38; SE, 1.74; *P* = .002).

**CONCLUSIONS AND RELEVANCE:**

In this study, the ASD PRS was associated with ASD traits among children from the general population. Genetic risks for ASD might be associated with delays in some neurodevelopmental domains, such as gross motor and receptive language skills.

## Introduction

Autism spectrum disorder (ASD) is among the most common neurodevelopmental disorders. It has been shown that genetic factors play an important role in the development of this disease, with a high heritability of approximately 80%.^1^ Both rare and common variants have been considered associated with ASD^2^; however, recent genome-wide association studies (GWASs) of ASD have reported several associated genomic loci with modest contributions of common variants to ASD.^3^ These variants can be used to generate measures called *polygenic risk scores* (PRSs), which are considered indicators of genetic liability for certain diseases or phenotypes. Although Rai et al^4^ demonstrated that an ASD PRS was associated with phenotypes relevant to ASD in adults from the general population, the contribution of the ASD PRS to ASD traits in children has not been well elucidated.

To search for early signs for ASD, previous research, including our own,^5^ examined possible neurodevelopmental delays in ASD and found that some specific domains, such as language and motor skills, were affected in individuals with ASD.^6^ However, the association of common genetic risk variants for ASD with affected neurodevelopmental stagnation requires further clarification.

Moreover, although 70% of individuals with ASD do not have intellectual disability,^7^ the diagnosis of ASD without intellectual disability remains a significant challenge. Recent genetic analyses found that common risk variants for ASD are associated with high IQ,^3^ and individuals with ASD and rare risk variants have high comorbidity with intellectual disability.^2^ Therefore, we examined whether the PRS for ASD was associated with neurodevelopmental delays at age 18 months, particularly in the motor and language domains, and with ASD traits at age 6 years in a populationbased birth cohort without intellectual disability. In addition, we conducted gene set enrichment analyses to obtain biological insights into ASD traits and neurodevelopmental progress derived from common genetic risk variants.

## Methods

### Participants

A total of 876 infants (438 [50.0%] boys) born between December 2007 and June 2011 in Japan were included. Recruitment procedures are fully described elsewhere.^5^ The study procedures were approved by the Hamamatsu University School of Medicine and University Hospital Ethics Committee, and written informed consent was obtained from each mother for her infant’s participation. This study followed the Strengthening the Reporting of Observational Studies in Epidemiology (STROBE) reporting guideline.

Overall, 66 individuals with intellectual disability (ie, full IQ <80 at age 4 years) were excluded. An additional 8 participants with parents of non-Japanese descent were removed from the study to minimize the potential effect of population stratification.

### Measurement

The Mullen Scales of Early Learning (MSEL) was used to evaluate neurodevelopmental progress at age 18 months. Module 3 of the Autism Diagnostic Observation Schedule 2 (ADOS-2) was used by certified raters to measure ASD traits at age 6 years, and the Wechsler Preschool and Primary Scale of Intelligence, Third Edition, was used by trained psychologists to measure full IQ at age 4 years.

### Genotyping, Quality Control, and Imputation

Genotyping was conducted using the Japonica array, designed for single-nucleotide polymorphism (SNP) genotyping specific to the Japanese population.^8^ Briefly, the quality controls retaining SNPs and participants were as follows: missing data for SNPs were less than 2%, pairwise identity by descent was less than 0.2, Hardy-Weinberg equilibrium for SNPs was *P* > 10^−6^, and minor allele frequency was greater than 0.01. A total of 76 individuals were removed from analysis because they were shown to be related by identity-by-descent analysis. Genotyping imputation was performed using BEAGLE 5.0 with phase 3 of the 1000 Genomes Project as a reference panel for the Japanese population.^9^ We excluded SNPs with an imputation INFO score of less than 0.8. We also excluded SNPs located within the major histocompatibility complex region because of high linkage disequilibrium in this region. The number of SNPs analyzed for the PRS was 5 606 655.

### Statistical Analysis

We generated the PRS with PRSice-2 using a recent Lundbeck Foundation Initiative for Integrative Psychiatric Research GWAS study for ASD as a discovery cohort.^3,10^ The summary GWAS data for ASD were obtained from the Psychiatric Genomics Consortium.^11^ To account for population stratification, 4 principle components calculated with PLINK 1.9 were used.^12^ The criterion for SNP clumping was a pairwise linkage disequilibrium of *R*^2^ less than 0.1 within 1-megabase windows. We calculated PRS scores with different *P* value thresholds, ranging from 5 × 10^−8^ to greater than .99, and best-fit PRSs were computed with highest *R*^2^ obtained from linear regression analysis. The *P* values for ASD traits and MSEL items were corrected using Bonferroni correction for 8 independent tests. Gene set enrichment analyses and exploratory analyses for searching compounds targeting enriched gene ontologies (GOs) were conducted using pRSet and gene2drug, respectively. Gene set collections were obtained from the Molecular Signatures Database, and the C5 GO gene sets were used for the analyses.^13^ The *P* values for PRSet were corrected by 10 000 permutation tests. The *P* values for gene2drug were corrected using Bonferroni corrections for 1309 compounds listed in the database. Data analysis took place from April to December 2019. Statistical significance was set at *P* < .05, and all tests were 2-tailed. All analyses were conducted in PRSice-2 and PLINK 1.9.

## Results

The summary of 876 participants’ characteristics is provided in Table 1. They had a mean (SD) gestational age at birth of 38.9 (1.6) weeks, 438 (50.0%) were boys, and 868 (99.1%) were Japanese. A total of 726 participants (373 [51.4%] boys) were analyzed.

The ASD PRS was associated with ASD traits measured by total scores on the ADOS-2 (*R*^2^ = 0.024; β, 0.71; SE, 0.25; corrected *P* = .03) and social affect (*R*^2^ = 0.027; β, 0.63; SE, 0.22; corrected *P* = .03) but not restricted repetitive behavior (*R*^2^ = 0.023; β, 0.19; SE, 0.07; corrected *P* = .06) (Table 2). The association of PRS for ASD with infant neurodevelopment was most pronounced in gross motor skills (*R*^2^ = 0.015; β, −1.25; SE, 0.39; corrected *P* = .01) and receptive language skills (*R*^2^ = 0.014; β, −1.19; SE, 0.39; corrected *P* = .02) measured by the MSEL (Table 2).

Gene set enrichment analyses found that several pathways, such as cell maturation (GO:0048469; *R*^2^ = 0.057; β, −5.28; SE, 1.40; corrected *P* < .001), adenylyl cyclase activity and cyclic adenosine monophosphate concentration (GO:0007193; *R*^2^ = 0.064; β, −5.30; SE, 1.30; corrected *P* < .001), regulation of amino acid transport (GO:0051955; *R*^2^ = 0.052; β, −5.03; SE, 1.44; corrected *P* = .001), and regulation of glutamate secretion (GO:0014048; *R*^2^ = 0.052; β, −5.82; SE, 1.68; corrected *P* = .001), were associated with ASD traits (Table 3). Gene set enrichment analyses for MSEL items identified many pathways associated with ASD PRS. For example, MSEL gross motor scores were associated with pathways associated with protein metabolism (eg, regulation of protein localization: GO:0032880; *R*^2^ = 0.020; β, 6.90; SE, 1.87; corrected *P* < .001; negative regulation of protein metabolic process: GO:0051248; *R*^2^ = 0.021; β, 8.82; SE, 2.37; corrected *P* < .001), and receptive language scores were associated with broader pathways (eg, establishment of RNA localization: GO:0051236; *R*^2^ = 0.022; β, −3.94; SE, 1.07; corrected *P* < .001; midbrain development: GO:0030901; *R*^2^ = 0.022; β, 7.17; SE, 1.91; corrected *P* < .001) (Table 4).

We compared the results of gene set analyses between ASD traits and associated neurodevelopmental domains. Among gene sets associated with ASD traits in our population, we found that the adenylate cyclase–inhibiting G protein–coupled receptor signaling pathway (GO:0007193) was associated with both gross motor and receptive language scores (*R*^2^ = 0.064; β, −5.30; SE, 1.30; corrected *P* < .001). Other gene sets commonly associated with ASD traits and MSEL gross motor scores included inflammation-associated gene sets, such as chemokine motif ligand 2 production (GO:0072567; *R*^2^ = 0.051; β, −6.04; SE, 1.75; corrected *P* = .001), regulation of monocyte differentiation (GO:0045655; *R*^2^ = 0.052; β, −6.63; SE, 1.90; corrected *P* = .001), and B-cell differentiation (GO:0030183; *R*^2^ = 0.051; β, 7.37; SE, 2.15; corrected *P* = .001) (Table 5). Interleukin 12 secretion (GO:0072610) was associated with ASD and gross motor skills (*R*^2^ = 0.047; β, −6.35; SE, 1.96; corrected *P* = .002). For receptive language scores, several gene sets associated with glutamatergic signaling, such as regulation of glutamate secretion (GO:0014048; *R*^2^ = 0.052; β, −5.82; SE, 1.68; corrected *P* = .001), ionotropic glutamate receptor signaling pathway (GO:0035235; *R*^2^ = 0.047; β, 3.54; SE, 1.09; corrected *P* = .001), and negative regulation of glutamate secretion (GO:0014050; *R*^2^ = 0.045; β, −5.38; SE, 1.74; corrected *P* = .002), were identified as common gene sets associated with ASD traits and MSEL receptive language scores (Table 5).

An exploratory analysis using the 10 most significant GOs enriched in ASD traits identified several compounds interacting with these gene sets. However, none of them remained significant after multiple testing corrections.

## Discussion

In this cohort study, we found that common genetic risk variants for ASD were associated with ASD traits in Japanese children without intellectual disability from the general population. The association of ASD PRS with ASD traits in the general population was consistent with a previous report,^4^ which showed that scores of social communication and repetitive behavior were higher among individuals with high (ie, >90th percentile) ASD PRSs. The outcomes used and the ages of participants in the previous report were slightly different from those of our study; however, it could be fair to say that ASD PRSs were associated with ASD traits in the general population.

We also found that these risk variants have a significant association with some domains of neurodevelopmental progress, such as gross motor and receptive language skill development in children from the general population without intellectual disability. Early detection and intervention for ASD is required; however, the mean patient age at ASD detection is generally between 3 and 4 years, which may miss the opportunity for effective intervention because accelerated brain development occurs between birth and the age of 4 years.^14^ Previous studies,^15,16^ including our own,^5^ have suggested that neurodevelopmental delays are relevant to ASD, and while ASD individuals without intellectual disability do not always manifest overall neurodevelopmental delays, our findings indicated that common genetic risks for ASD were associated with stagnation in specific neurodevelopmental domains, such as gross motor and receptive language skills. Thus, it might be important for clinicians and caregivers to focus on these neurodevelopmental domains to detect and consider interventions for the future development of ASD in children without intellectual disability.

A study by Clarke et al^17^ found that the ASD PRS was positively associated with cognitive ability in the general adult population, although the results were not replicated in the second cohort. Similarly, Grove et al^3^ reported that higher IQ was associated with ASD PRS. These findings may seem inconsistent with our findings; however, they could be explained by differences in the study populations (ie, adult vs child) and the outcomes measured (ie, cognitive function vs neurodevelopmental progress). There is a possibility that the ASD PRS was associated with neurodevelopmental trajectory and not with final cognitive achievements. Further longitudinal studies investigating the association of ASD PRS with cognitive development are warranted.

Several gene sets were identified as enriched in ASD traits in our population. Many of these gene sets were associated with glutamatergic signaling and neurotransmitter signaling, which is consistent with a previous gene set enrichment analysis of individuals with ASD.^18^ Excitability and inhibitory balance has long been implicated in the etiology of ASD,^19^ and it could be that this system has also been involved in the development of ASD traits in the general population.

The adenylate cyclase–inhibiting G protein–coupled receptor signaling pathway (GO:0007193) was strongly associated with ASD traits, gross motor skills, and receptive language progress. This gene set includes many genes encoding neurotransmitter receptors, such as *DRD2* (OMIM 126450), *DRD3* (OMIM 126451), *GABBR1* (OMIM 603540), *GRM2* (OMIM 604099), and *HTR1A* (OMIM 109760), which are strongly implicated in various psychiatric diseases, including ASD,^20^ and in neurodevelopment.^21^

Another important finding was that immune systems, such as regulation of monocyte differentiation (GO:0030224) and interleukin 12 secretion (GO:0072610), might have roles in the development of ASD traits and gross motor progress. Many studies have suggested that immune systems are involved in the pathophysiology of ASD,^22^ and a 2019 cohort study^23^ showed that inflammation was associated with delayed gross motor progress at age 30 months. Although it is still unknown whether delays in gross motor progress are a direct risk of ASD or vice versa, molecules related to immune systems, such as cytokines, might be a useful for biomarker for ASD and gross motor progress. Given that immunomodulatory therapies have been proposed for ASD, this could have beneficial outcomes for these phenotypes.^22^

Furthermore, we found that gene sets associated with glutamate signaling were closely involved in ASD traits and receptive language skills in our study sample. Although they are still under development, several glutamate signaling modulators have been tested for ASD^24^ based on the findings from various clinical and animal studies.^24,25^ It has also been reported that delayed progress in receptive language is observed in individuals with a mutation in the *GRIN2A* gene (OMIM 13853).^26^ Taken together, glutamate signaling is associated with both ASD traits and early language development.

### Limitations

This study has limitations. First, we targeted a representative sample of the general population, and thus, caution is needed when applying the findings to individuals with clinically diagnosed ASD. Second, because there has been no large GWAS of ASD conducted using East Asian populations, we used a study with a white population as a discovery cohort, which may partially explain the low *R*^2^ in our PRS analyses.

## Conclusions

In this study, the ASD PRS was associated with ASD traits among children in the general population. These genetic risks were associated with specific neurodevelopmental delays. Immune systems and glutamate signaling might be common pathways associated with ASD traits and neurodevelopmental progress.

## Figures and Tables

**Table 1.** Characteristics of 876 Participating Infants and Their Parents

Characteristic	No. (%)
Birth weight, mean (SD), g	2935.1 (444.3)
Age at birth, mean (SD)	
Gestational, wk	38.9 (1.6)
Paternal, y	33.5 (5.7)
Maternal, y	29.3 (5.2)
Household income, mean (SD), millions of ¥	6.1 (2.7)
Sex	
Boys	438 (50.0)
Girls	438 (50.0)
Race/ethnicity	
Japanese	868 (99.1)
Mixed^a^	
White	5 (0.5)
Latino	3 (0.3)
Size for gestational age, percentile	
<10th	785 (89.6)
10th-100th	91 (10.4)
Ratio of placenta to birth weight, percentile	
<10th	164 (18.7)
10th-100th	712 (81.3)
Paternal education, y	
<12	65 (7.4)
≥12	811 (92.6)
Maternal education, y	
<12	38 (4.3)
≥12	838 (95.7)

a Mixed indicates that 1 parent was not of Japanese descent.

**Table 2.** Association of Autism Spectrum Disorder Polygenic Risk Score With Autism Spectrum Disorder Traits

Trait	*P* Value Threshold	SNPs, No.	*R* ^2^	β (SE)	*P* Value^a^
ADOS-2					
Social affect	.005	3492	0.027	0.63 (0.22)	.03
Restricted repetitive behavior	.006	3002	0.023	0.19 (0.07)	.06
Total	.006	3492	0.024	0.71 (0.25)	.03
MSEL					
Gross motor skills	.01	4493	0.015	−1.25 (0.39)	.01
Fine motor skills	5.00 × 10^−8^	3	0.007	0.84 (0.38)	.22
Receptive language development	.008	4512	0.014	−1.19 (0.39)	.02
Expressive language development	.008	5997	0.004	−0.66 (0.37)	.59
Visual reception	.02	8144	0.004	−0.60 (0.38)	.90

Abbreviations: ADOS-2, Autism Diagnostic Observation Schedule 2; MSEL, Mullen Scales of Early Learning; SNP, single-nucleotide polymorphism.

a *P* values were corrected with Bonferroni corrections.

**Table 3.** Gene Sets Enriched in Autism Spectrum Disorder Traits

Gene Set	*P* Value Threshold	SNPs, No.	*R* ^2^	β (SE)	*P* Value^a^
Cell maturation	.02	311	0.057	−5.28 (1.40)	<.001
Adenylate cyclase-inhibiting G protein-coupled receptor signaling pathway	.04	381	0.064	−5.30 (1.30)	<.001
Regulation of amino acid transport	.04	278	0.052	−5.03 (1.44)	.001
Regulation of glutamate secretion	.03	142	0.052	−5.82 (1.68)	.001
Neuron maturation	.01	165	0.052	−6.52 (1.87)	.001
Ionotropic glutamate receptor signaling pathway	.02	335	0.047	3.54 (1.09)	.001
Locomotion	.01	3642	0.045	1.06 (0.34)	.002
Negative regulation of glutamate secretion	.04	122	0.045	−5.38 (1.74)	.002
Regulation of cell communication by electrical coupling involved in cardiac conduction	.18	302	0.046	−5.26 (1.66)	.002
Negative regulation of organic acid transport	.04	189	0.047	−5.125 (1.60)	.002

Abbreviation: SNP, single-nucleotide polymorphism.

a *P* values were corrected with 10 000 permutation tests.

**Table 4.** Gene Sets Enriched in MSEL Gross Motor and Receptive Language Scores

MSEL Item	Gene Set	*P* Value Threshold	SNPs, No.	*R* ^2^	β (SE)	*P* Value^a^
Gross motor skills	Neutral lipid metabolic process	.48	174	0.031	−5.09 (1.10)	<.001
	Neutral lipid biosynthetic process	.07	23	0.022	−8.26 (2.14)	<.001
	Regulation of protein localization	.0005	22	0.020	6.90 (1.87)	<.001
	Negative regulation of protein metabolic process	.0003	17	0.021	8.82 (2.37)	<.001
	Regulation of blood pressure	.006	25	0.020	−7.90 (2.18)	<.001
	Negative regulation of cellular amide metabolic process	.02	39	0.020	7.21 (1.98)	<.001
Receptive language skills	Establishment of RNA localization	.22	172	0.022	−3.94 (1.07)	<.001
	Nucleobase containing compound transport	.22	193	0.021	−3.71 (1.02)	<.001
	Intracellular protein transport	.002	31	0.020	4.95 (1.39)	<.001
	Midbrain development	.26	61	0.022	7.17 (1.91)	<.001
	Response to dietary excess	.31	25	0.020	−10.38 (2.94)	<.001
	RNA localization	.22	188	0.020	−3.70 (1.04)	<.001
	Male gamete generation	.004	24	0.018	7.42 (2.20)	<.001
	Sertoli cell differentiation	.34	13	0.020	17.10 (4.82)	<.001

Abbreviations: MSEL, Mullen Scales of Early Learning; SNP, single-nucleotide polymorphism.

a *P* values were corrected with 10 000 permutation tests

**Table 5.** List of Gene Sets Commonly Enriched in Autism Spectrum Disorder Traits and MSEL Items

MSEL Item	Gene Set	*P* Value Threshold	SNPs, No.	*R* ^2^	β (SE)	*P* Value^a^
Gross motor skills	Adenylate cyclase-inhibiting G protein-coupled receptor signaling pathway	.04	38	0.064	−5.30 (1.30)	<.001
	Muscle cell development	.008	44	0.052	3.91 (1.12)	<.001
	Chemokine motif ligand 2 production	.14	17	0.051	−6.04 (1.75)	.001
	Regulation of monocyte differentiation	.14	18	0.052	−6.63 (1.90)	.001
	B-cell differentiation	.001	11	0.051	7.37 (2.15)	.001
	Monocyte activation	.14	12	0.048	−6.61 (2.01)	.001
	Interleukin 12 secretion	.14	13	0.047	−6.35 (1.96)	.002
Receptive language skills	Adenylate cyclase-inhibiting G protein-coupled receptor signaling pathway	.04	38	0.064	−5.30 (1.30)	<.001
	Regulation of amino acid transport	.04	27	0.052	−5.03 (1.44)	.001
	Regulation of glutamate secretion	.03	14	0.052	−5.82 (1.68)	.001
	Neuron maturation	.01	16	0.052	−6.52 (1.87)	.001
	lonotropic glutamate receptor signaling pathway	.02	33	0.047	3.54 (1.09)	.001
	Negative regulation of glutamate secretion	.04	12	0.045	−5.38 (1.74)	.002

Abbreviations: MSEL, Mullen Scales of Early Learning; SNP, single-nucleotide polymorphism.

a *P* values were corrected with 10 000 permutation tests.

## Key Points

### Question

Are genetic risks for autism spectrum disorder (ASD) also associated with neurodevelopment in infancy and ASD traits among children in the general population?

### Findings

In this cohort study of 734 infants from the Hamamatsu Birth Cohort for Mothers and Children, genetic risks for ASD were associated with ASD traits at age 6 years. The polygenic risk score for ASD was associated with delays in gross motor and receptive language skills at age 18 months.

### Meaning

Genetic risks for ASD are associated with ASD traits in the general population and neurodevelopmental progress among infants as young as 18 months.
